# Small Colony Variants of *Staphylococcus aureus* and Pacemaker-related Infection

**DOI:** 10.3201/eid0910.030200

**Published:** 2003-10

**Authors:** Harald Seifert, Hilmar Wisplinghoff, Petra Schnabel, Christof von Eiff

**Affiliations:** * University of Cologne, Cologne, Germany; † University of Münster Hospital and Clinics, Münster, Germany

## Abstract

We describe the first known case of a device-related bloodstream infection caused by *Staphylococcus aureus* small colony variants. Recurrent pacemaker-related bloodstream infection within a 7-month period illustrates the poor clinical and microbiologic response to prolonged antimicrobial therapy in a patient infected with this *S. aureus* subpopulation.

Infections caused by *Staphylococcus aureus* range from mild skin infections to acute life-threatening diseases such as pneumonia, osteomyelitis, and endocarditis. However, *S. aureus* may also cause a chronic disease with persistent and recurrent infections. Skin and soft tissue infections, chronic osteomyelitis, and persistent infections in patients with cystic fibrosis have been associated with small colony variants, a naturally occurring subpopulation of the species *S. aureus* (*1*–*6*). *S. aureus* small colony variants are characterized as electron transport deficient bacteria because of their auxotrophism to hemin or menadione or are recognized as thymidine-dependent. These variants produce very small, mostly nonpigmented and nonhemolytic colonies. In addition, they also demonstrate various other features that are atypical for *S. aureus,* including reduced coagulase production, failure to use mannitol, and increased resistance to aminoglycosides and cell-wall active antibiotics (*3*–*10*). Furthermore, the ability of these variants to persist intracellularly within nonprofessional phagocytes has been described (*3*,*5*,*11*). Because of their fastidious growth characteristics and unusual morphologic appearance, small colony variants present a challenge both to the microbiologist and the clinician, often resulting in misidentification and misinterpretation (*1*,*2*,*7*,*8*). Prerequisite for recovering and identifying these variants is the application of extended conventional culture and identification techniques (*3*,*5*,*8*). We report the first case of a pacemaker-related bloodstream infection caused by *S. aureus* small colony variants. This case illustrates the poor clinical and microbiologic response to prolonged antimicrobial therapy in patients infected with these variants.

## Case Report

A 63-year old man was transferred to our facility with the presumptive diagnosis of endocarditis related to a pacemaker-lead infection. Past medical history included hypertension, coronary artery disease, and noninsulin-dependent diabetes mellitus. A VVI (ventricular ventricular inhibited) pacemaker had been implanted for treatment of sick sinus syndrome 9 years earlier. Six weeks before admission, this device had been removed because of a pocket infection after blunt trauma with dislocation of the device and perforation of the skin. Specimens for microbiologic culture were not obtained at this time. The pacemaker leads were left in place, a gentamicin-containing sponge was applied to the infection site, and a new pacemaker was implanted on the other side of the chest. Four weeks later, the patient sought treatment at the local hospital for a high fever (39.7°C) and chills and a subcutaneous abscess with oxacillin-susceptible *S. aureus* at the primary insertion site*.* After surgical drainage, antimicrobial therapy was initiated with intravenous cefuroxime. The remaining pacemaker leads were partially cut but not completely removed. Ten days later, spiking fever and chills unresponsive to the administration of meropenem and vancomycin developed, and the patient was transferred to our medical center for pacemaker ablation. The physical examination did not indicate auscultation abnormalities or stigmata of endocarditis. Laboratory studies were unremarkable except a C-reactive protein (CRP) level of 170 mg/L (normal value <8 mg/L) and a blood sedimentation rate of 79 mm/h. Multiple blood cultures taken on admission remained negative. Transesophageal echocardiography did not show vegetations or other evidence of endocarditis. On hospital day 6, the new pacemaker was completely removed by percutaneous ablation as were the remaining leads of the old device. Only the tip of the pacemaker lead remained fixed in the myocardium, and surgical removal involving extracorporal circulation was not attempted. The patient’s condition improved rapidly, CRP level returned to normal, and on hospital day 32, the patient was transferred to the local hospital to complete a 6-week course of intravenous vancomycin and rifampin as empirical antistaphylococcal therapy. Before transfer, the daily vancomycin dose had been reduced to 250 mg twice a day after an elevated vancomycin serum level. Eight days later, the patient was readmitted with recurrent high fever. Blood cultures taken on readmission were again negative. After the vancomycin dose was increased to 500 mg every 12 hours, the patient promptly became afebrile. Antimicrobial therapy was discontinued after the patient had completed a 10-week course of vancomycin and rifampin. Three days later, the patient again had spiking fever. After 6 to 48 hours of incubation, four sets of blood cultures obtained on four consecutive days yielded nonpigmented and nonhemolytic staphylococci, initially identified on the basis of a negative tube coagulation test and the API ID 32 Staph system (bioMérieux, Marcy-L’Etoile, France) as coagulase-negative staphylococci, susceptible to oxacillin (MIC 0.5 μg/mL) and vancomycin (MIC 1.0 μg/mL) but resistant to rifampin (MIC >32 μg/mL). However, the colony morphologic findings were suggestive of small colony variants of *S. aureus*, confirmed by polymerase chain reaction amplification of the *nuc* and *coa* genes as well as by determination of the stain’s auxotrophy for hemin. The patient responded promptly to flucloxacillin, 4 g intravenously three times a day. After another 6-week course of parenteral therapy, antimicrobial therapy was discontinued, and the patient was discharged (CRP 7 mg/L, ESR 35 mm/h); he was readmitted after 6 days with chills and high fever. Antimicrobial therapy with intravenous flucloxacillin was resumed and followed by immediate defervescence. Three blood cultures taken on readmission were again positive with *S. aureus* small colony variants. Clonal identity of all isolates was demonstrated by pulsed-field gel electrophoresis of bacterial DNA (data not shown). A transesophageal echocardiogram showed the residual tip of the pacemaker lead fixed in the myocardial septum without vegetations. The remaining device was finally removed by open-heart surgery with use of cardiopulmonary bypass. Microbiologic culture of the pacemaker electrode performed at a different institution yielded abundant growth of staphylococci that were misidentified as *S. warneri,* showing the same biochemical profile as the previously isolated bacteria as determined by the ID 32 Staph system. The patient recovered completely and was discharged on the 10th postoperative day after a total hospital course of 7 months.

## Conclusions

*S. aureus* small colony variants have been implicated in persistent and recurrent infections that give a poor clinical and bacteriologic response to standard antimicrobial therapy in patients with abscess, chronic osteomyelitis, and bronchopulmonary infections, particularly after prolonged exposure to antibiotics (*1*–*6*). Bloodstream infection related to an implantable intravascular device with this *S. aureus* variant has not been reported before. These phenotypic variants are characterized by their fastidious growth and atypical colony morphologic findings on routine media, making recovery as well as correct identification difficult for microbiologic laboratories (*3*,*5*,*8*). The ability to interrupt electron transport and to form a variant subpopulation affords *S. aureus* a number of survival advantages, including the ability of this subpopulation to persist intracellularly within nonprofessional phagocytes (*11*,*12*). The intracellular position may shield small colony variants from host defenses and decrease exposure to antibiotics (*3*,*5*,*11*). *S. aureus* small colony variants can be selected by gentamicin in vitro and in vivo as shown in patients with osteomyelitis after gentamicin bead placement (*4*,*12*). Chuard et al. demonstrated that, in contrast to their normal phenotype parental strain, *S. aureus* small colony variants that were attached to fibronectin-coated coverslips were highly resistant to cell-wall–active antimicrobial agents such as oxacillin and vancomycin (*13*).

In our case, findings suggest that *S. aureus* small colony variants might have been selected from the parent strain population with a normal phenotype after exposure to the locally applied aminoglycoside or to the prolonged administration of vancomycin. Continually positive blood cultures with the same strain as demonstrated by molecular typing and the presumable persistence of these organisms on the pacemaker lead tip may partly be explained by the poor effectiveness of vancomycin and flucloxacillin against these slow-growing organisms that were adhering to the remaining foreign body and the ability of these variants to persist intracellularly (*7*,*8*,*13*).

This case adds to the spectrum of persistent and relapsing infections attributed to *S. aureus* small colony variants and emphasizes that these variants may also play a role in intravascular device–related infections. It also illustrates that complete removal of any foreign body material is essential for the complete cure of prosthetic intravascular device–related *S. aureus* infection. Laboratories should be particularly alert for *S. aureus* small colony variants when samples are submitted from patients who have received long-term antimicrobial therapy, especially if the infection is unusually persistent or recurrent.

## References

[1] Proctor RA, Bates DM, McNamara PJ. Electron transport deficient *Staphylococcus aureus* small-colony variants as emerging pathogens. In: Scheld WM, Craig WA, Hughes JM, editors. Emerging infections 5. Washington: ASM Press; 2001.

[2] Proctor RA, van Langevelde P, Kristjansson M, Maslow JN, Arbeit RD. Persistent and relapsing infections associated with small-colony variants of *Staphylococcus aureus.* Clin Infect Dis. 1995;20:95–102. 7727677 10.1093/clinids/20.1.95

[3] von Eiff C, Becker K, Metze D, Lubritz G, Hockmann J, Schwarz T, Intracellular persistence of *Staphylococcus aureus* small-colony variants within keratinocytes: a cause for antibiotic treatment failure in a patient with Darier’s disease. Clin Infect Dis. 2001;32:1643–7. 10.1086/320519 11340539

[4] von Eiff C, Bettin D, Proctor RA, Rolauffs B, Lindner N, Winkelmann W, Recovery of small colony variants of *Staphylococcus aureus* following gentamicin bead placement for osteomyelitis. Clin Infect Dis. 1997;25:1250–1. 10.1086/516962 9402396

[5] Kahl B, Herrmann M, Schulze-Everding A, Koch HG, Becker K, Harms E, Persistent infection with small colony variant strains of *Staphylococcus aureus* in patients with cystic fibrosis. J Infect Dis. 1998;177:1023–9. 9534977 10.1086/515238

[6] Seifert H, von Eiff C, Fätkenheuer G. Fatal case due to methicillin-resistant *Staphylococcus aureus* small colony variants in an AIDS patient. Emerg Infect Dis. 1999;5:450–3. 10.3201/eid0503.990319 10341185 PMC2640760

[7] Proctor RA, Kahl B, von Eiff C, Vaudaux PE, Lew DP, Peters G. Staphylococcal small colony variants have novel mechanisms for antibiotic resistance. Clin Infect Dis. 1997;27(Suppl 1):S68–74. 10.1086/514906 9710673

[8] Proctor RA, Peters G. Small colony variants in staphylococcal infections: diagnostic and therapeutic implications. Clin Infect Dis. 1998;27:419–23. 10.1086/514706 9770133

[9] Kahl BC, Belling G, Reichelt R, Herrmann M, Proctor RA, Peters G. Thymidine-dependent small-colony variants of *Staphylococcus aureus* exhibit gross morphological and ultrastructural changes consistent with impaired cell separation. J Clin Microbiol. 2003;41:410–3. 10.1128/JCM.41.1.410-413.2003 12517881 PMC149606

[10] Baumert N, von Eiff C, Schaaff F, Peters G, Proctor RA, Sahl HG. Physiology and antibiotic susceptibility of *Staphylococcus aureus* small colony variants. Microb Drug Resist. 2002;8:253–60. 10.1089/10766290260469507 12523621

[11] Vaudaux P, Francois P, Bisognano C, Kelley WL, Lew DP, Schrenzel J, Increased expression of clumping factor and fibronectin-binding proteins by hemB mutants of *Staphylococcus aureus* expressing small colony variant phenotypes. Infect Immun. 2002;70:5428–37. 10.1128/IAI.70.10.5428-5437.2002 12228267 PMC128368

[12] Balwit JM, van Langevelde P, Vann JM, Proctor RA. Gentamicin-resistant menadione and hemin auxotrophic *Staphylococcus aureus* persist within cultured endothelial cells. J Infect Dis. 1994;170:1033–7. 7930701 10.1093/infdis/170.4.1033

[13] Chuard C, Vaudaux PE, Proctor RA, Lew DP. Decreased susceptibility to antibiotic killing of a stable small colony variant of *Staphylococcus aureus* in fluid phase and on fibronectin-coated surfaces. J Antimicrob Chemother. 1997;39:603–8. 10.1093/jac/39.5.603 9184359

